# Supplementary material for: Dynamics of Physiological Properties and Endophytic Fungal Communities in the Xylem of Aquilaria sinensis (Lour.) with Different Induction Times
Source: J Fungi (Basel). 2024 Aug 9;10(8):562. doi: 10.3390/jof10080562 (PMC11355071; doi:10.3390/jof10080562)
Supplement: Supplementary file 1 [file jof-10-00562-s001.zip › jof-3123709-supplementary.pdf]

**Dynamics of Physiological Properties and Endophytic Fungal Communities in the Xylem of *Aquilaria sinensis* (Lour.) with Different Induction Times**

## Supplementary Materials

**Table S1**. The characteristic of sequence, base and goods coverage after sequence for each xylem sample. 0M, 3M, 6M and 9M represent before pre-induction, the third month, the sixth month and the ninth month after artificial induction, respectively.

| Sample | Raw Sequence Number | Base Number | Mean Length | Min Length | Max Length | Effective Sequences | Goods Coverage |
|---|---|---|---|---|---|---|---|
| 0M-1 | 187039 | 52368736 | 279.99 | 49 | 387 | 174412 | 0.9998 |
| 0M-2 | 230074 | 62826763 | 273.07 | 42 | 389 | 190923 | 0.9999 |
| 0M-3 | 160919 | 52667155 | 327.29 | 42 | 391 | 12950 | 0.9992 |
| 0M-4 | 160593 | 48667217 | 303.05 | 42 | 377 | 67519 | 0.9998 |
| 3M-1 | 229511 | 71057881 | 309.61 | 50 | 391 | 226038 | 0.9995 |
| 3M-2 | 186038 | 57436503 | 308.74 | 42 | 389 | 182629 | 0.9995 |
| 3M-3 | 241852 | 69881718 | 288.94 | 42 | 388 | 238130 | 0.9996 |
| 3M-4 | 195691 | 60957292 | 311.5 | 50 | 391 | 192321 | 0.9995 |
| 6M-1 | 151069 | 48037719 | 317.99 | 42 | 391 | 56248 | 0.9997 |
| 6M-2 | 162257 | 48798230 | 300.75 | 42 | 379 | 158043 | 0.9995 |
| 6M-3 | 173382 | 50860077 | 293.34 | 42 | 380 | 168157 | 0.9995 |
| 6M-4 | 205599 | 60115923 | 292.39 | 42 | 389 | 201384 | 0.9996 |
| 9M-1 | 201593 | 59755588 | 296.42 | 42 | 387 | 196279 | 0.9996 |
| 9M-2 | 174364 | 48307771 | 277.05 | 45 | 378 | 171110 | 0.9995 |
| 9M-3 | 133453 | 43998180 | 329.69 | 42 | 388 | 10687 | 0.9953 |
| 9M-4 | 32398 | 10011979 | 309.03 | 45 | 391 | 15130 | 0.9970 |

**Table S2**. Variations in the relative abundance of dominant orders with induction time. Values in the table were mean ± SD (n=4). 0M, 3M, 6M and 9M represent pre-induction, the third month, the sixth month and the ninth month after artificial induction, respectively. * $P < 0.05$, ** $P < 0.01$, ns > 0.05.

| **Dominant Orders** | **Induction Time** | | | | **ANOVA** |
|---|---|---|---|---|---|
| | 0M | 3M | 6M | 9M | F-value |
| Polyporales | 5.05±3.07 | 64.41±9.02 | 1.26±0.12 | 5.10±3.07 | 15.02** |
| Eurotiales | 7.90±3.80 | 1.53±0.04 | 58.56±16.04 | 13.25±6.18 | 7.52** |
| Chaetothyriales | 4.24±2.44 | 7.51±2.58 | 16.20±9.96 | 24.55±5.97 | 3.83* |
| Calosphaeriales | 10.20±7.93 | 7.38±1.90 | 0.38±0.13 | 5.46±3.13 | 3.83* |
| Xylariales | 0.04±0.03 | 0.50±0.10 | 0.92±0.37 | 4.91±2.68 | 8.69** |
| Chaetosphaeriales | 0.03±0.02 | 0.33±0.02 | 0.21±0.07 | 1.18±0.42 | 8.75** |
| Dothideales | 3.75±2.09 | 0.007±0.001 | 0.37±0.36 | 0.13±0.08 | 4.47* |
| Sordariales | 7.29±3.29 | 0.08±0.01 | 0.74±0.67 | 0.58±0.23 | 4.88* |
| Agaricales | 1.38±1.37 | 0.04±0.01 | 0.41±0.38 | 0.26±0.13 | 0.45ns |
| Annulatascales | 3.45±3.34 | 0.07±0.01 | 0.04±0.01 | 0.26±0.11 | 0.98ns |
| Conioscyphales | 10.50±7.62 | 6.02±1.93 | 0.33±0.11 | 2.25±0.69 | 3.09ns |
| Diaporthales | 0.20±0.20 | 1.06±0.51 | 0.15±0.05 | 5.51±4.37 | 3.22ns |
| Glomerellales | 1.55±1.45 | 0.006±0.001 | 0.26±0.26 | 0.19±0.18 | 0.83ns |
| Hypocreales | 11.85±4.22 | 1.66±0.32 | 12.50±11.68 | 12.23±6.90 | 1.08ns |
| Magnaporthales | 1.09±0.95 | 0.002±0.001 | 0.003±0.002 | 0.18±0.12 | 1.40ns |
| Ophiostomatales | 0 | 0.030±0.004 | 0.23±0.15 | 1.93±1.75 | 1.39ns |
| Pezizales | 1.63±0.74 | 0.003±0.002 | 0.29±0.27 | 0.29±0.23 | 3.06ns |
| Pleosporales | 5.94±1.37 | 7.47±6.00 | 1.28±0.15 | 5.53±3.15 | 1.13ns |
| Saccharomycetales | 6.09±2.73 | 0.49±0.03 | 1.07±0.36 | 2.21±1.20 | 2.46ns |
| Tremellales | 1.04±0.86 | 0.13±0.09 | 1.01±0.54 | 0.88±0.48 | 0.68ns |

**Table S3**. Variations in the relative abundance of dominant genera with induction time

| Dominant Genera | Induction Time | | | | ANOVA |
|---|---|---|---|---|---|
| | 0M | 3M | 6M | 9M | F-value |
| Candelabrochaete | 4.98±3.08 | 64.41±9.02 | 1.01±0.14 | 5.03±3.08 | 15.77** |
| Talaromyces | 0.03±0.02 | 1.44±0.03 | 57.05±16.95 | 12.28±6.11 | 22.08** |
| Jattaea | 10.20±7.93 | 7.38±1.90 | 0.38±0.13 | 5.46±3.13 | 3.83* |
| Rhinocladiella | 0.08±0.07 | 0.19±0.02 | 0.15±0.05 | 9.91±5.99 | 4.95* |
| Gibberella | 8.48±3.16 | 0.09±0.02 | 0.33±0.26 | 0.44±0.21 | 7.76** |
| Kurtzmanomyces | 0.17±0.12 | 0.36±0.08 | 0.13±0.04 | 7.66±3.94 | 10.10** |
| Phialemoniopsis | 0.005±0.004 | 0.49±0.09 | 0.34±0.06 | 4.76±2.73 | 9.71** |
| Aureobasidium | 3.75±2.09 | 0.007±0.001 | 0.37±0.36 | 0.13±0.08 | 4.47* |
| Nigrograna | 0 | 0.46±0.01 | 0.36±0.12 | 1.68±0.97 | 3.61* |
| Pyrigemmula | 0.03±0.02 | 0.33±0.02 | 0.21±0.07 | 1.09±0.40 | 8.48** |
| Chaetomium | 1.67±0.94 | 0.003±0.0001 | 0.006±0.004 | 0.013±0.008 | 4.05* |
| Conioscypha | 10.50±7.62 | 6.02±1.93 | 0.34±0.11 | 2.25±0.69 | 3.09ns |
| Trichoderma | 0.19±0.18 | 1.35±0.32 | 11.66±11.11 | 3.26±1.14 | 1.50ns |
| Exophiala | 2.91±1.32 | 5.05±1.02 | 15.92±9.92 | 13.55±7.76 | 2.20ns |
| Aspergillus | 7.82±3.78 | 0.091±0.003 | 0.92±0.69 | 0.92±0.71 | 3.19ns |
| Fusarium | 1.38±0.82 | 0.149±0.006 | 0.22±0.08 | 7.78±7.19 | 1.35ns |
| Italiomyces | 0.20±0.19 | 1.06±0.52 | 0.15±0.05 | 5.51±4.37 | 3.20ns |
| Ascitendus | 3.45±3.34 | 0.07±0.01 | 0.04±0.01 | 0.26±0.11 | 0.98ns |
| Pichia | 2.64±1.88 | 0.12±0.01 | 0.09±0.03 | 0.49±0.25 | 2.62ns |
| Cladophialophora | 0.05±0.03 | 2.14±1.58 | 0.08±0.03 | 0.71±0.25 | 2.70ns |
| Sporothrix | 0 | 0.030±0.004 | 0.23±0.15 | 1.93±1.75 | 1.39ns |
| Plectosphaerella | 1.48±1.47 | 0.004±0.001 | 0.20±0.20 | 0.19±0.18 | 0.62ns |
| Echria | 1.74±1.57 | 0.001±0.0001 | 0.02±0.001 | 0 | 1.61ns |
| Roussoella | 1.01±1.01 | 0.021±0.002 | 0.015±0.006 | 0.42±0.32 | 0.76ns |
| Issatchenkia | 1.39±1.35 | 0.003±0.001 | 0.006±0.003 | 0.009±0.004 | 1.12ns |
| Sarocladium | 1.30±1.27 | 0.001±0.0001 | 0 | 0.001±0.001 | 1.13ns |
| Neoascochyta | 1.03±1.03 | 0 | 0.002±0.001 | 0.007±0.007 | 0.99ns |
| Arxiella | 1.02±0.97 | 0.001±0.001 | 0.001±0.001 | 0.013±0.007 | 1.23ns |

**Table S4**. The main central taxa in the networks under the different induction times

| Induction Time | Central Taxa (higher connected nodes) | | |
|---|---|---|---|
| 0M | Sebacinales | Helotiales | Cystofilobasidiales |
| 3M | Pleosporales | Orbiliales | Botryosphaeriales |
| 6M | Hypocreales | Pleosporales | Sordariales |
| 9M | Polyporales | Conioscyphales | Pleosporales/ Pezizale |

**Table S5**. Conditional effects of physio-biochemical properties on endophytic fungal diversity and potential function in redundancy analysis. SOD, POD and MDA represent superoxide dismutase, peroxidase and malondialdehyde, respectively. * $P < 0.05$, ** $P < 0.01$, ns $P > 0.05$.

| **Explanatory** | **Fungal Diversity** | | **Explanatory** | **Potential Function** | |
|---|---|---|---|---|---|
| **Variables** | Explains (%) | F-value | **Variables** | Explains (%) | F-value |
| Starch | 61.6 | 22.5** | SOD | 32.3 | 6.7** |
| Soluble sugar | 15.1 | 8.5* | POD | 7.2 | 1.5ns |
| MDA | 0.2 | 0.1ns | MDA | 3.7 | 0.8ns |
| POD | <0.1 | <0.1ns | Starch | 3.0 | 0.6ns |
| SOD | <0.1 | <0.1ns | Soluble sugar | 1.7 | 0.3ns |

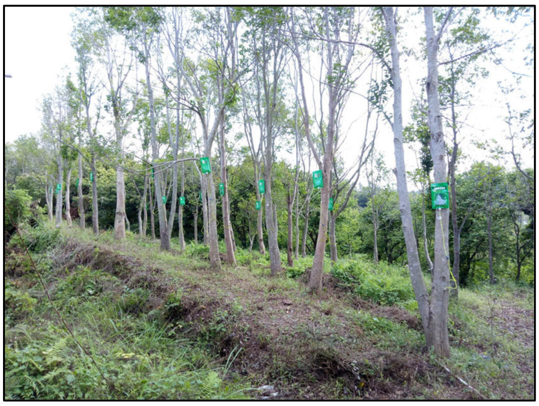

**Figure S1**. Artificial induction with an inorganic salt solution in *Aquilaria sinensis* plantation

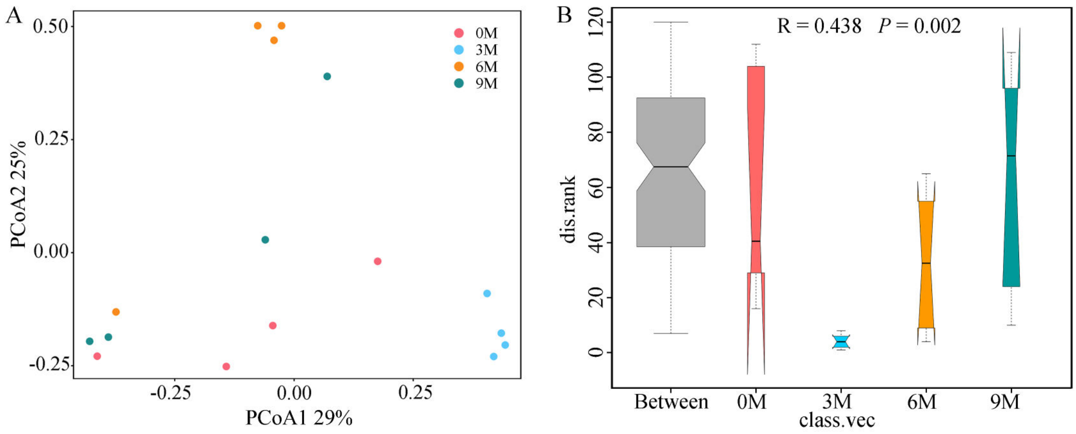


**Figure S2**. Discrepancy (A) and ANOSIM (B) of endophytic fungal community function predicted based on FUNGuild. 0M, 3M, 6M and 9M represent pre-induction, the third month, the sixth month and the ninth month after artificial induction, respectively.

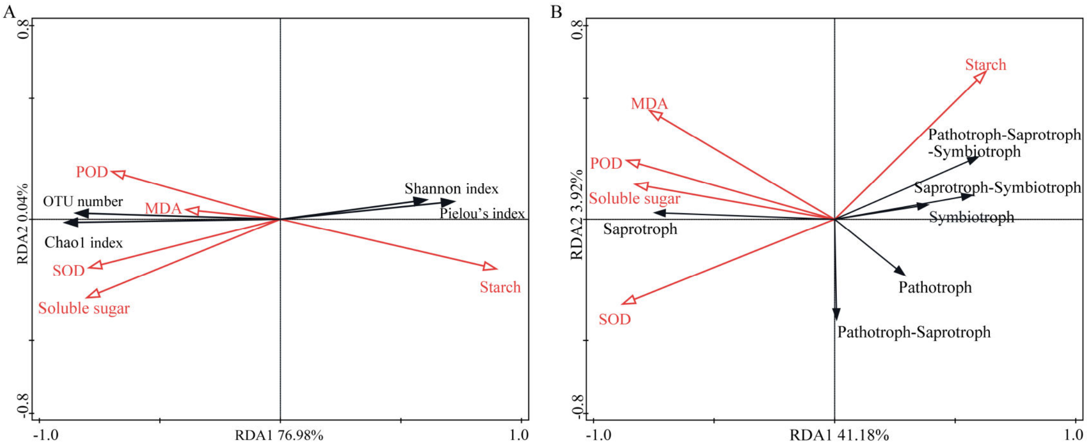


**Figure S3**. Redundancy analysis (RDA) between physio-biochemical parameters and fungal diversity (A) and potential function (B). SOD, POD and MDA represent superoxide dismutase, peroxidase and malondialdehyde, respectively.
